# Supplementary material for: The predictive value of PRDM2 in solid tumor: a systematic review and meta-analysis
Source: PeerJ. 2020 Apr 29;8:e8826. doi: 10.7717/peerj.8826 (PMC7195840; doi:10.7717/peerj.8826)
Supplement: File S1 [file peerj-08-8826-s001.docx]

**The rationale for conducting the meta-analysis**

Cancer has long been considered a catastrophic public health problem due to its high mortality rates. In 2018, cancer has become the second leading cause of death worldwide, with an estimate of 9.6 million death. In order to reduce its mortality, cancer control should be enforced. One example of cancer control is an early diagnosis of cancer through the use of tumor biomarkers. Unfortunately, the implementation of tumor biomarkers in the clinical setting is still lacking. Hence, further research to identify novel biomarkers should be performed.

Positive Regulatory/Su(var)3-9, Enhancer-of-zeste and Trithorax Domain 2 (PRDM2) is a tumor suppressor gene (TSG) that belongs to the nuclear histone/protein methyltransferase superfamily. Many studies have reported *PRDM2* downregulation in several types of cancer, including those that exhibit high incidence and mortality. Thus, it is evident that *PRDM2* might have a potential role as a biomarker in cancer, especially in solid tumors. However, the potential of *PRDM2* as a diagnostic biomarker is still unclear. Therefore, we conducted a systematic review and meta-analysis that addressed this issue.

**The contribution that the meta-analysis makes to knowledge in light of previously published related reports, including other meta-analyses and systematic reviews**

In our study, we have successfully generated the first meta-analysis that investigated the potential of *PRDM2* downregulation as a diagnostic biomarker in solid tumor. The pooled analysis suggested that *PRDM2* gene expression is decreased in solid tumor (RR 4.29, 95% CI 2.58 – 7.13, P < 0.00001), hinting that *PRDM2* could be used as a biomarker in solid tumors. The summary sensitivity and specificity of decreased *PRDM2* gene expression in solid tumor is 84% (95% CI 39-98%) and 86% (95% CI 71-94%), respectively. This result is also in favor of *PRDM2* downregulation as a potential diagnostic biomarker. However, the confidence interval for *PRDM2* downregulation is wide, suggesting that there is marked imprecision. This was later confirmed on TSA whereby the cumulative Z-curve failed to cross the significance boundary and did not reach the required number of studies which is 7743. Hence, it can be concluded that the use of *PRDM2* as a diagnostic biomarker in solid tumors is still inconclusive.

Although our present study could not fully prove the potential of *PRDM2* downregulation as a diagnostic biomarker due to its imprecision, it is important to highlight that these results can potentially improve with the addition of new studies. This has been proven by our TSA results whereby the line representing the cumulative Z-curve did not cross the futility boundary. Thus, further research is required.
