# Supplementary material for: The predictive value of PRDM2 in solid tumor: a systematic review and meta-analysis
Source: PeerJ. 2020 Apr 29;8:e8826. doi: 10.7717/peerj.8826 (PMC7195840; doi:10.7717/peerj.8826)
Supplement: File S2 [file peerj-08-8826-s002.docx]

| **Section/topic** | **#** | **Checklist item** | **Reported on page #** |
| --- | --- | --- | --- |
| **TITLE** | | |  |
| Title | 1 | Identify the report as a systematic review, meta-analysis, or both. | 1 |
| Comment |  | Lline 1-2: The predictive value of PRDM2 in solid tumor: a **systematic review and meta-analysis** |  |
| **ABSTRACT** | | |  |
| Structured summary | 2 | Provide a structured summary including, as applicable: background; objectives; data sources; study eligibility criteria, participants, and interventions; study appraisal and synthesis methods; results; limitations; conclusions and implications of key findings; systematic review registration number. | 1-2 |
| Comment |  | Background (line 21-23): **Many studies have reported the presence of Positive Regulatory/Su(var)3-9, Enhancer-of-zeste and Trithorax Domain 2 (PRDM2) downregulation in cancer. However, its potential as a diagnostic biomarker is still unclear**.  Objective (line 28-31): This study **aims to assess the relationship between *PRDM2* downregulation and solid tumor, its relationship with clinicopathological data, and its potential as a diagnostic biomarker.** This study also **aims to evaluate the quality of the studies, data reliability and confidence in cumulative evidence.**  Systematic review registration number (line 34): **CRD42019132156.**  Methods (line 34-38): **PRISMA was used as a guideline** to conduct this review. A **comprehensive electronic search was performed from inception to June 2019 in Pubmed, Cochrane Library, ProQuest, EBSCO and ScienceDirect**. **Studies were screened and included studies were identified based on the criteria made**. Finally, **data synthesis and quality assessment were conducted.**  Results (line 39-43): There is a **significant relationship between *PRDM2* downregulation with solid tumor (RR 4.29, 95% CI 2.58 – 7.13, P < 0.00001)**. The **overall sensitivity and specificity of *PRDM2* downregulation in solid tumors is 84% (95% CI 39-98%) and 86% (95% CI 71-94%), respectively**. There is a **low risk of bias** for the studies used. TSA results suggested the **presence of marked imprecision**. The **overall quality of evidence for this study is very low**.  Limitations (line 46-47): A **major source of limitation** in this study is the **small number of studies**.  Conclusions (line 48-53): ***PRDM2* is downregulated in solid tumor**. **The relationship between *PRDM2* downregulation and clinicopathological data is still inconclusive**. Although the sensitivity and specificity of *PRDM2* downregulation are imprecise, its high values, in addition to the evidence presented that confirmed ***PRDM2* downregulation in solid tumor suggested that it might still have a potential to be used as a diagnostic biomarker**. In order to further strengthen these findings, **more research regarding *PRDM2* in solid tumors are encouraged.** |  |
| **INTRODUCTION** | | |  |
| Rationale | 3 | Describe the rationale for the review in the context of what is already known. | 2 |
| Comment |  | Line 71-79: **Positive Regulatory/Su(var)3-9, Enhancer-of-zeste and Trithorax Domain 2 (PRDM2*)* is a tumor suppressor gene (TSG**) that belongs to the nuclear histone/protein methyltransferase superfamily. *PRDM2* gene products are **involved in DNA-binding and transcription factor binding-activities**, implicating its **role in carcinogenesis** (Zhang et al. 2015). Furthermore, studies have also reported ***PRDM2* downregulation in cancers that exhibit high incidence and mortality**. However, the **potential of *PRDM2* as a diagnostic biomarker is still unclear.** |  |
| Objectives | 4 | Provide an explicit statement of questions being addressed with reference to participants, interventions, comparisons, outcomes, and study design (PICOS). | 2-3 |
| Comment |  | Line 81-84: Therefore, we **performed a systematic review and meta-analysis that investigated the relationship between *PRDM2* expression level and solid tumor, as well as its potential as a diagnostic biomarker**. If there is sufficient data, we will also investigate if there is any **correlation between *PRDM2* expression level with clinicopathological data.** |  |
| **METHODS** | | |  |
| Protocol and registration | 5 | Indicate if a review protocol exists, if and where it can be accessed (e.g., Web address), and, if available, provide registration information including registration number. | 3 |
| Comment |  | Line 88-90: A protocol of this study is registered at the **International Prospective Register of Systematic Reviews (PROSPERO)** with the following registration number: **CRD42019132156 (**[**https://www.crd.york.ac.uk/prospero/display_record.php?RecordID=132156**](https://www.crd.york.ac.uk/prospero/display_record.php?RecordID=132156)**).** |  |
| Eligibility criteria | 6 | Specify study characteristics (e.g., PICOS, length of follow-up) and report characteristics (e.g., years considered, language, publication status) used as criteria for eligibility, giving rationale. | 3 |
| Comment |  | Line 96-97: A comprehensive electronic search was done in PubMed, Cochrane Library, ProQuest, EBSCO and ScienceDirect **from inception to July 2019**  Line 109-117: **A study is included** **if** it meets the following criteria: (**1) The study used human subjects; (2) The study investigated the relationship between *PRDM2* expression level and solid tumor through the use of gene expression analysis; (3) The study used histopathological examination as a comparator; (4) The study is a clinical trial or cross-sectional study. A study is excluded if: (1) The study does not have a control group (people without cancer or non-cancer specimens); (2) The study did not use an appropriate or did not state the gene expression analysis method used; (3) The expression level of *PRDM2* in the study is not clearly stated or unquantifiable; (4) The study is a review, case series, conference abstracts, in vitro or in vivo study. (5) The study is not written in English.** |  |
| Information sources | 7 | Describe all information sources (e.g., databases with dates of coverage, contact with study authors to identify additional studies) in the search and date last searched. | 3 |
| Comment |  | Line 96-97: A comprehensive electronic search was done in **PubMed, Cochrane Library, ProQuest, EBSCO and ScienceDirect** **from inception to July 2019** |  |
| Search | 8 | Present full electronic search strategy for at least one database, including any limits used, such that it could be repeated. | 3 |
| Comment |  | Line 97-100: **(PRDM2 OR RIZ OR RIZ1 OR RIZ2 OR KMT8 OR KMT8A OR MTB-ZF OR HUMHOXY1) AND (Cancer OR Cancers OR Malignant OR Malignancy OR Malignancies OR Neoplasm OR Neoplasms OR Neoplasia OR Neoplasias OR Tumor OR Tumors OR Tumour OR Tumours).** |  |
| Study selection | 9 | State the process for selecting studies (i.e., screening, eligibility, included in systematic review, and, if applicable, included in the meta-analysis). | 3 |
| Comment |  | Line 102-104: Duplicates were removed, and **screening was performed based on the title and abstract of the study. Probable or included studies were identified and assessed for eligibility according to the criteria above.** |  |
| Data collection process | 10 | Describe method of data extraction from reports (e.g., piloted forms, independently, in duplicate) and any processes for obtaining and confirming data from investigators. | 3-4 |
| Comment |  | Line 102: All of the search outputs were exported into the **EndNote software**. **Duplicates were removed**  Line 123-124: In the case of **missing data**, the **authors will be contacted via email to request access** to those missing data. |  |
| Data items | 11 | List and define all variables for which data were sought (e.g., PICOS, funding sources) and any assumptions and simplifications made. | 3-4 |
| Comment |  | Line 120-123: The included studies were then analyzed further and the following informations are extracted: **First author, publication year, country of origin, age, gender, race, type of cancer, cancer differentiation state, stage of cancer, type of control, number of cases and controls, gene expression analysis method, *PRDM2* expression level and conclusion of the study.** |  |
| Risk of bias in individual studies | 12 | Describe methods used for assessing risk of bias of individual studies (including specification of whether this was done at the study or outcome level), and how this information is to be used in any data synthesis. | 4-5 |
| Comment |  | Line 160-162: **Quality of evidence** will be assessed using **Quality Assessment of Diagnostic Accuracy Studies - 2 (QUADAS-2)**  Line 166-167: **Grading of Recommendations, Assessment, Development, and Evaluations (GRADE) was used to evaluate the confidence in cumulative evidence.** |  |
| Summary measures | 13 | State the principal summary measures (e.g., risk ratio, difference in means). | 4 |
| Comment |  | Line 129-130: **Sensitivity and specificity** of PDRM2 were assessed in order to elucidate the potential of *PRDM2* expression level as a diagnostic biomarker in solid tumor.  Line 131-133: **Risk ratio (RR) with a 95% confidence interval (CI)** was used to determine the relationship between *PRDM2* expression level and risk of cancer, as well as the relationship between *PRDM2* expression level and clinicopathological data. |  |
| Synthesis of results | 14 | Describe the methods of handling data and combining results of studies, if done, including measures of consistency (e.g., I^2^) for each meta-analysis. | 4 |
| Comment |  | Line 133-135: If **heterogeneity is present**, **Random Effects Model (REM)** **will be used.** However, **if heterogeneity is absent, Fixed Effects Model (FEM) will be used instead**.  Line 137-138: **Cochrane’s Q test (chi-squared test) and Higgins I^2^ statistics** were used to assess for the presence of heterogeneity statistically. |  |

| **Section/topic** | **#** | **Checklist item** | **Reported on page #** |
| --- | --- | --- | --- |
| Risk of bias across studies | 15 | Specify any assessment of risk of bias that may affect the cumulative evidence (e.g., publication bias, selective reporting within studies). | 4 |
| Comment |  | Line 145-146: **Funnel plot and Deek’s test will be used to assess publication bias** when the number of included studies is at least 10.  Line 147-148: **If publication bias is found, the trim and fill method will be used to correct this bias**  Line 159-160: Thus, a **trial sequential analysis (TSA) was performed using TSA software** in order to determine the required information size. |  |
| Additional analyses | 16 | Describe methods of additional analyses (e.g., sensitivity or subgroup analyses, meta-regression), if done, indicating which were pre-specified. | 4 |
| Comment |  | Line 140-143: **Meta-regression and subgroup analysis** will be conducted when there are at least 10 studies used in the meta-analysis. The possible causes of heterogeneity are: **Age, gender, ethnicity, country of origin, type of cancer, cancer differentiation state, stage of cancer and genotyping method.**  Line 150-153: Furthermore, **sensitivity analysis was performed to elucidate the effect and stability of a single study on the pooled estimates by deleting one study at a time**. Additionally, **sensitivity analysis was also conducted to compare the pooled estimates using odds ratio (OR) and RR, as well as using REM and FEM**. |  |
| **RESULTS** | | |  |
| Study selection | 17 | Give numbers of studies screened, assessed for eligibility, and included in the review, with reasons for exclusions at each stage, ideally with a flow diagram. | 5 and Figure 1 |
| Comment |  | Line 172-174: Using the search method previously described above, a total of **58 potential studies were identified.** Out of these 58 studies, **52 were excluded due to the studies being irrelevant, in vitro and/or in vivo, used unsuitable methods, written in non-English, or is a review.** |  |
| Study characteristics | 18 | For each study, present characteristics for which data were extracted (e.g., study size, PICOS, follow-up period) and provide the citations. | 5, Table 1 and 2 |
| Comment |  | Line 181-192: **The studies that were eligible for systematic review were published from 1999 to 2015. There was a total of 314 samples of solid tumors and 225 controls obtained from patients in China (Dong et al. 2012; Ge et al. 2015; Tan et al. 2018), Japan (Akahira et al. 2007), Sweden (Geli et al. 2005) and United States of America (Jiang et al. 1999). All of these six studies are cross-sectional studies. The solid tumors included in this study are ovarian cancer (Akahira et al. 2007), esophageal squamous cell carcinoma (Dong et al. 2012),** **renal cell carcinoma (RCC) (Ge et al. 2015), pheochromocytoma (Geli et al. 2005), abdominal paraganglioma (Geli et al. 2005), hepatoma (Jiang et al. 1999), lung squamous cell carcinoma (LSCC) (Tan et al. 2018) and lung adenocarcinoma (LAC) (Tan et al. 2018). Out of these six studies, one used immunohistochemistry (IHC) only (Akahira et al. 2007), three used reverse transcription-polymerase chain reaction (RT-PCR) only (Ge et al. 2015; Geli et al. 2005; Jiang et al. 1999) and two used both IHC and RT-PCR (Dong et al. 2012; Tan et al. 2018).** |  |
| Risk of bias within studies | 19 | Present data on risk of bias of each study and, if available, any outcome level assessment (see item 12). | 7, Table 3-4 |
| Comment |  | Line 243-244: In general, the **quality of the included studies was superior**, ensuring the reliability of our systematic review and meta-analysis.  Line 258-259: Overall, we have **very low confidence in the pooled estimates** obtained for our meta-analysis. |  |
| Results of individual studies | 20 | For all outcomes considered (benefits or harms), present, for each study: (a) simple summary data for each intervention group (b) effect estimates and confidence intervals, ideally with a forest plot. | 5-6, Table 1 |
| Comment |  | Line 199-207: **All six studies concluded that *PRDM2* gene expression is significantly decreased in solid tumor compared to control, with the P-value ranging from <0.05 to <0.001 using CI 95%.** **Akahira et al. (2007)** stated that there was a **significant correlation between *PRDM2* downregulation with cancer grade (P<0.0345) and stage (P<0.0153) in ovarian cancer**. On the other hand, **Ge et al. (2015)** stated otherwise, concluding that there was **no significant relationship between RCC with tumor progression (P=0.19)**. A study by **Geli et al. (2005)** reported that **decreased *PRDM2* gene expression was not correlated significantly with gender and tumor size, but was found to be weakly correlated with younger age (Spearman rank-order correlations; R=0.4).** Other clinicopathological data were either absent or not investigated in the studies. |  |
| Synthesis of results | 21 | Present results of each meta-analysis done, including confidence intervals and measures of consistency. | 6, Figures 2, 6-7 and S1 |
| Comment |  | Line 215-216: The pooled analysis suggested that *PRDM2* gene expression is decreased in solid tumor (**RR 4.29, 95% CI 2.58 – 7.13, P < 0.00001**; Fig. 2).  Line 224-227: As demonstrated in the summary receiver operating characteristic (SROC) curve (Fig. 7), the **summary sensitivity and specificity of decreased *PRDM2* gene expression in solid tumor is 84% (95% CI 39-98%) and 86% (95% CI 71-94%), respectively.** |  |
| Risk of bias across studies | 22 | Present results of any assessment of risk of bias across studies (see Item 15). | 6-7, Figures 8 and S2 |
| Comment |  | Line 228-231: However, the confidence interval for *PRDM2* downregulation is wide**, suggesting that there is marked imprecision. This was later confirmed on TSA (Fig. 8).** In Fig. 8, the line representing **the cumulative Z-curve failed to cross the significance boundary and did not reach the required number of studies which is 7743.**  Line 255-256: As for publication bias, since the number of included studies is <10, **publication bias could not be evaluated.**  Line 269-271: Meta-regression, **funnel plot and Deek’s test were not performed due to the small number of studies obtained. Due to the inability to confirm the presence of publication bias, we also could not perform trim and fill method.** |  |
| Additional analysis | 23 | Give results of additional analyses, if done (e.g., sensitivity or subgroup analyses, meta-regression [see Item 16]). | 6-7, Figures 3-5 |
| Comment |  | Line 219-220: **All three sensitivity analyses did not have meaningful differences, proving that our results are stable.**  Line 269-270: **Meta-regression**, funnel plot and Deek’s test **were not performed due to the small number of studies obtained.**  Line 271-272: Since our results indicated that there was **no heterogeneity in the studies used,** **a subgroup analysis was not required.** |  |
| **DISCUSSION** | | |  |
| Summary of evidence | 24 | Summarize the main findings including the strength of evidence for each main outcome; consider their relevance to key groups (e.g., healthcare providers, users, and policy makers). | 7-8 |
| Comment |  | Line 274-275: In line with previous studies, our results demonstrated that ***PRDM2* downregulation occurs in solid tumor.**  Line 277-279: Another notable difference is **the inconclusive results linking *PRDM2* downregulation with cancer stage and grade** even though *PRDM2* downregulation has been associated with cancer progression (Sun et al. 2011).  Line 290-291: Thus, the **use of *PRDM2* downregulation as a diagnostic biomarker is still inconclusive.**  Line 303-307: Hence, indirectness is present, and this could lead to overestimation of sensitivity and specificity, resulting in the downgrading of the quality of evidence (Schmidt & Factor 2013). As discussed before, imprecision is present, and publication bias could not be assessed, leading to further downgrading. **Together, these three domains led to the downgrading of the quality of evidence from high to very low.** |  |
| Limitations | 25 | Discuss limitations at study and outcome level (e.g., risk of bias), and at review-level (e.g., incomplete retrieval of identified research, reporting bias). | 8 |
| Comment |  | Line 311-320: **Limitations of our study are the lack of RCTs** as part of our included studies which made it difficult to evaluate the internal validity of our results (Carlson & Morrison 2009). As mentioned before, our study also **lacks clinicopathological data** in order to assess the potential of *PRDM2* further. Interestingly, none of the included studies investigated *PRDM2* gene expression in the same type of solid tumor. Hence, we were **unable to evaluate in which type of solid tumor is *PRDM2* downregulation most suitable to be used as a biomarker.** Furthermore, there was **no standardised baseline among studies.** Another limitation of this study involves the issue of **only using studies written in English,** leading to the **possibility of language bias.** **Most of the individual studies have a wide confidence interval and inconclusive TSA results,** indicating there is insufficient knowledge about the effect and that further research should be done. |  |
| Conclusions | 26 | Provide a general interpretation of the results in the context of other evidence, and implications for future research. | 9 |
| Comment |  | Line 325-330: In conclusion, our review demonstrated **that *PRDM2* gene expression is decreased or downregulated in solid tumor.** Due to insufficient data, we are **unable to determine the relationship between *PRDM2* downregulation and clinicopathological data.** Although the sensitivity and specificity of *PRDM2* downregulation are imprecise, its high values, in addition to the evidence presented that confirmed *PRDM2* downregulation in solid tumor, suggested that it **might still have a potential to be used as a diagnostic biomarker.**  Line 331-337: Thus, we **suggest more research to be conducted, especially those with RCT as their design**. **More study is urgently needed to determine a standardised baseline for *PRDM2* downregulation level.** We would also recommend **more research regarding the relationship between *PRDM2* gene expression with clinicopathological data** to further evaluate the potential of *PRDM2* gene expression in solid tumor. Finally, we suggest **a new systematic review and meta-analysis to be done in order to renew the findings of our study.** |  |
| **FUNDING** | | |  |
| Funding | 27 | Describe sources of funding for the systematic review and other support (e.g., supply of data); role of funders for the systematic review. | Funding statement |

*From:*  Moher D, Liberati A, Tetzlaff J, Altman DG, The PRISMA Group (2009). Preferred Reporting Items for Systematic Reviews and Meta-Analyses: The PRISMA Statement. PLoS Med 6(7): e1000097. doi:10.1371/journal.pmed1000097

For more information, visit: **www.prisma-statement.org**.
