# Supplementary material for: The predictive value of PRDM2 in solid tumor: a systematic review and meta-analysis
Source: PeerJ. 2020 Apr 29;8:e8826. doi: 10.7717/peerj.8826 (PMC7195840; doi:10.7717/peerj.8826)
Supplement: Table S2 [file peerj-08-8826-s004.docx]

**Supplemental Table 2: QUADAS-2 scoring of included studies**

| **Studies** | **Patient Selection** | | | **Applicability Concerns** | **Index Test** | | **Applicability Concerns** | **Reference Standard** | | **Applicability Concerns** | **Flow and Timing** | | | | **Score** | **Study Rating** |
| --- | --- | --- | --- | --- | --- | --- | --- | --- | --- | --- | --- | --- | --- | --- | --- | --- |
|  | **Consecutive/Random sampling?** | **Case-control avoided?** | **Inappropriate exclusion avoided?** | **Patients match review question?** | **Index test results independent?** | **Threshold pre-specified?** | **Index test match review question?** | **Reference standard likely to correctly classify?** | **Reference standard results independent?** | **Target condition match review question?** | **Appropriate interval between tests?** | **All patients receive reference standard?** | **All patients receive the same reference standard?** | **All patients included in analysis?** |  |  |
| Akahira 2007 | Y | Y | Y | Y | Y | Y | Y | Y | Y | Y | Y | Y | Y | Y | 14 | Low risk of bias |
| Dong 2012 | Y | Y | Y | Y | Y | Y | Y | Y | Y | Y | Y | Y | Y | N | 13 | Low risk of bias |
| Ge 2015 | Y | Y | Y | Y | U | Y | Y | Y | Y | Y | Y | Y | Y | N | 12 | Low risk of bias |
| Geli 2005 | Y | Y | Y | Y | U | Y | Y | Y | Y | Y | Y | Y | Y | Y | 13 | Low risk of bias |
| Jiang 1999 | U | U | U | Y | U | Y | Y | Y | Y | Y | Y | Y | Y | Y | 10 | Low risk of bias |
| Tan 2018 | Y | Y | Y | Y | U | Y | Y | Y | Y | Y | Y | Y | Y | Y | 13 | Low risk of bias |

N = No. U = Unclear. Y = Yes. Note: N = 0. U = 0. Y = 1. High risk of bias if <10. Low risk of bias if >=10.
