# Supplementary figures and images for: The predictive value of PRDM2 in solid tumor: a systematic review and meta-analysis
Source: PeerJ. 2020 Apr 29;8:e8826. doi: 10.7717/peerj.8826 (PMC7195840; doi:10.7717/peerj.8826)

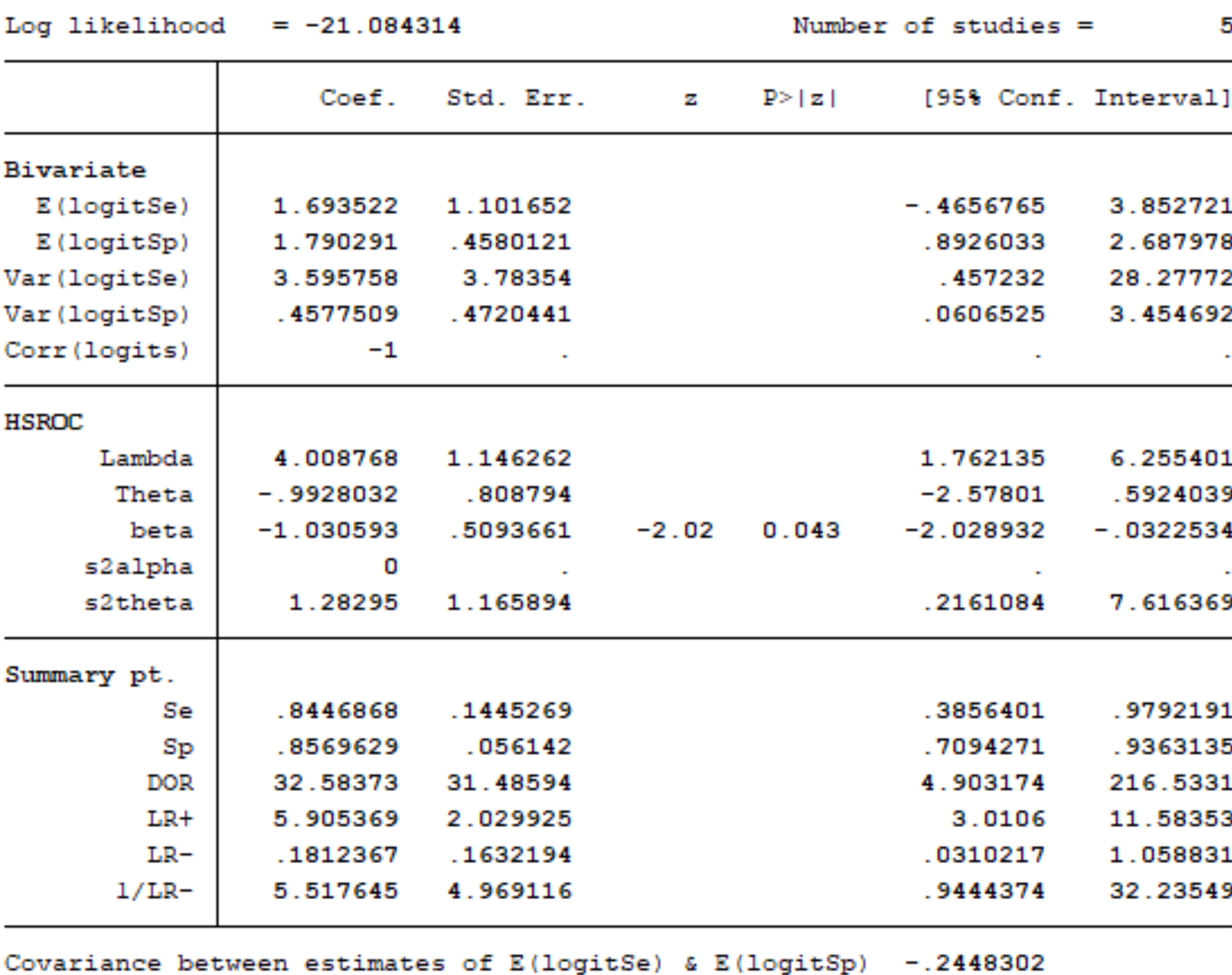

Supplement: Figure S1 [file peerj-08-8826-s005.png]

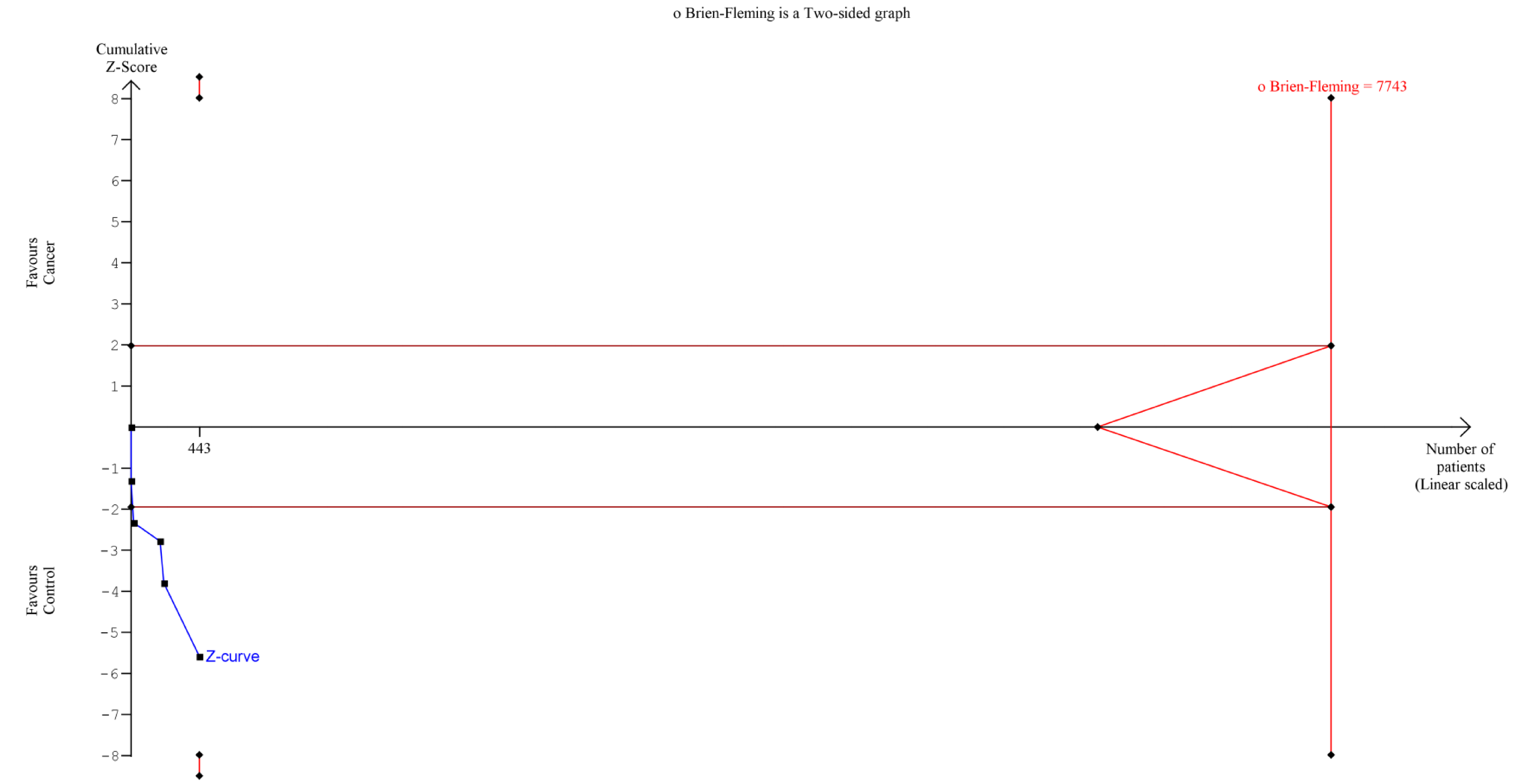

Supplement: Figure S2 [file peerj-08-8826-s006.png]
